# Exploring Genetic Link of Residual Ridge Resorption in Completely Edentulous Individuals: A Prospective Case-Control Clinical Study

**DOI:** 10.7759/cureus.64039

**Published:** 2024-07-07

**Authors:** Mahendirakumar Nagarajan, Vijitha Dayasekaran, Kavitha Jayavel, Merlin Jayaraj, Sreeja Chellaswamy, Krishnaraj Ranganathan, Sriram Kaliamoorthy, Sugirtha Chellapandi, Radhika Baskaran, Agila Elumalai

**Affiliations:** 1 Department of Prosthodontics and Crown and Bridge, Government Dental College and Hospital, Cuddalore, IND; 2 Department of Periodontics and Implantology, Government Dental College and Hospital, Cuddalore, IND; 3 Department of Oral and Maxillofacial Pathology, Chettinad Dental College and Research Institute, Chennai, IND; 4 Department of Dentistry, Vinayaka Mission's Medical College and Hospital, Vinayaka Mission's Research Foundation (DU), Karaikal, IND; 5 Department of Periodontics, Chettinad Dental College and Research Institute, Chennai, IND

**Keywords:** prosthodontics, polymorphism, genetic, alveolar resorption, alveolar process

## Abstract

Background: Residual ridge resorption presents obstacles in prosthodontic treatment, affecting denture stability and the success of dental implants. Genetic elements, specifically the single nucleotide polymorphism (SNP) 1772C>T variant within the hypoxia-inducible factor 1 subunit alpha (HIF-1α) gene, are hypothesized to contribute to residual ridge resorption progression. Nevertheless, its impact remains insufficiently investigated, especially within the context of South Indian populations. We sought to investigate the connection between SNP 1772C>T and residual ridge resorption (RRR) among fully edentulous individuals, considering demographic factors, genotyping methodologies, and statistical evaluations.

Methods: In a prospective case-control study, we recruited 100 completely edentulous participants from South India. Participants were categorized based on alveolar ridge height. Saliva samples were non-invasively collected for DNA extraction, and polymerase chain reaction-restriction fragment length polymorphism (PCR-RFLP) analysis was employed to determine genotype distribution using the HphI restriction enzyme. The statistical evaluations comprised the utilization of chi-square and Fisher's exact tests.

Results: We observed no significant variations in genotype distributions between the case and control cohorts (CT: p=0.24; CC: p=0.65; TT: p=0.30). The heterozygous genotype CT was prevalent in both groups.

Conclusions: Although we did not observe significant associations between SNP 1772C>T and RRR, our findings imply a genetic predisposition to residual ridge resorption that warrants further exploration. Variations in genetic susceptibility across ethnicities and the influence of other genetic variants on residual ridge resorption require additional investigation. This study lays the groundwork for personalized prosthodontic care by highlighting the potential of genetic analysis in routine dental practice to improve treatment strategies.

## Introduction

In prosthodontic practice, maintaining the structural integrity of residual ridges is crucial for the efficacy of dental interventions. Significant ridge resorption in edentulous individuals poses complications such as ill-fitting dentures and the risk of implant complications [1]. After tooth removal, a cascade of inflammatory agents guide the formation of clots, the growth of granulation tissue, and the regeneration of bone. Despite initial signs of wound healing, ongoing destructive remodeling results in the resorption of the alveolar ridge across the lifespan of an individual [2].

Understanding residual ridge resorption's intricate pathogenesis is challenging, attributed to anatomical, metabolic, and mechanical factors. The unique composition of the residual ridge, formed during tooth extraction wound healing, suggests that genetic regulatory factors influencing bone quality and quantity contribute to residual ridge resorption [3,4]. In oral wound healing, hypoxia-induced post-tooth extraction is crucial. Hypoxia-inducible factor-1 (HIF-1) regulating oxygen-dependent genes, particularly the alpha subunit (HIF-1α), plays a vital role [5]. Acknowledging the significance and genetic variability of HIF-1α, the research focuses on a prevalent single nucleotide polymorphism (SNP), 1772C>T, within the HIF-1α gene [6].

Despite being thoroughly studied across various medical conditions like malignancy, heart disease, lung disease, and diabetes, along with its potential effects on athletes, the relationship between SNP 1772C>T and residual ridge resorption remains unexplored. This study seeks to fill gaps in understanding by examining the correlation between a particular genetic alteration in the HIF-1α gene and the occurrence of significantly resorbed residual mandibular jaw bone. Our emphasis is on a specific demographic of patients to address a void in population-specific investigations. Our main objective is to comprehend the relationship between SNP 1772C>T and the occurrence of accelerated residual ridge resorption. Additionally, we explore the practical application of this genetic marker, which can be easily obtained from a simple saliva sample in a dental clinic. This marker has the potential to serve as a tool for predicting the anticipated resorption of the alveolar ridge before therapeutic intervention.

## Materials and methods

This case-control study was carried out at the Department of Prosthodontics, Government Dental College and Hospital, Cuddalore, India, for the duration of two years. This study was aimed to explore the relationship between the SNP 1772 C>T found in the hypoxia-inducible factor 1 subunit alpha gene and alveolar jaw bone resorption in completely edentulous male and female patients from South India, an area where such associations are not extensively studied. Unrelated individuals who have been edentulous for at least two years were included in the study. Subjects with systemic conditions affecting bone health such as diabetes and those with a history of alveolar bone augmentation were excluded from the study.

**Figure 1:**
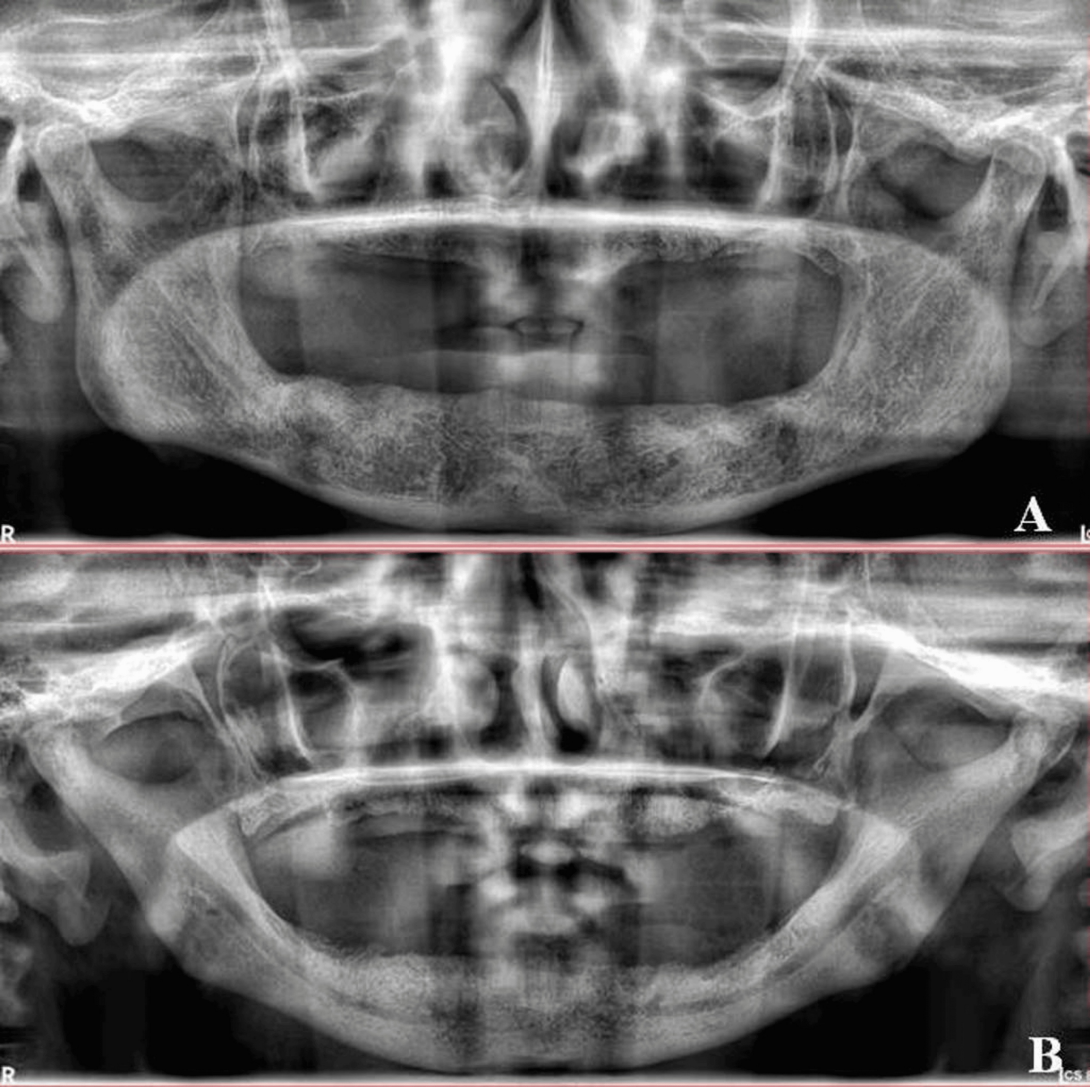
Orthopantomogram (OPG) images for control group type I (A) and case group type IV edentulous mandibular ridges (B).

Digital panoramic radiographs were employed to evaluate the condition and height of the alveolar ridge. Following the American College of Prosthodontics (ACP) classification protocol, patients were then categorically divided into two groups based on the minimum vertical residual alveolar ridge height. The ACP classification defines type I as having a residual ridge bone height of 21 mm or more, type II as having a residual ridge bone height between 16 mm and 20 mm, type III as having a residual ridge bone height between 11 mm and 15 mm, and type IV as having a residual ridge bone height of 10 mm or less [7]. The case group (Group A) comprised patients with severe resorption in the mandibular residual ridge (type III and type IV ridges). In contrast, the control group (Group B) consisted of patients with a well-developed lower jaw bone ridge with very minimal or no signs of resorption (type I and type II ridges) (Figures 1A, 1B).

Ethical approval was obtained from the Institutional Ethical Committee of Government Dental College and Hospital, Cuddalore, India (#IHEC/865/2022, dated: 25/05/2022). Salivary samples were collected through convenience sampling from patients who met the aforementioned inclusion and exclusion criteria. To determine the average occurrence of the 1772C>T SNP in the HIF-1α gene, a sample size of 100 patients was calculated and divided into two groups. This calculation was carried out with an alpha error of 0.05 to achieve a study power of 80%, this was performed using Epi-Info 7 software (Atlanta, GA: Centers for Disease Control and Prevention).

In genomic studies, blood cells are typically the primary and conventional source, but their collection is invasive. An alternative non-invasive source for genomic DNA is the buccal mucosal exfoliated squamous cells found in saliva. In the current study, selected participants were instructed to refrain from consuming foods and fluids for at least 30 minutes before the saliva sample collection [8]. The collected samples were refrigerated immediately to maintain their properties and DNA content until they were transported for further processing, ensuring their integrity was maintained. The salivary samples were transported to the lab and suspended in phosphate-buffered saline (PBS) and underwent centrifugation at 12,000 RPM for 5 minutes at 4°C. Afterward, the supernatant was removed and the resultant pellet was utilized for subsequent analysis. The DNA extraction procedure involved the application of the QIAamp DNA Blood Mini Kit (Hilden, Germany: QIAGEN) following the centrifugation instructions provided in the manufacturer's protocol.

The human HIF-1α gene resides on chromosome 14 (14q21-q24) and comprises 15 exons [9]. The particular SNP of interest is located more specifically in exon 12. In this investigation, the appropriate forward and reverse primers were employed to amplify the exon 12 segment to ensure the effective digestion of polymerase chain reaction (PCR) products, the HphI restriction endonuclease from ThermoFisher Scientific, USA, was chosen. In the PCR reactions, 100 ng of DNA served as the template. The PCR mixture utilized a 2X red dye master mix (amplicon). The PCR conditions consisted of an initial denaturation phase at 94°C for 4 minutes, followed by 35 cycles of denaturation at 94°C for 30 seconds, annealing and extension at 58°C for 2 minutes, and 72°C for 30 seconds. Lastly, a final extension step was executed at 72°C for 5 minutes.

Agarose gel electrophoresis was employed to separate the PCR products based on size. The separated PCR products were visualized using agarose gel electrophoresis, and their sizes were compared against a suitable DNA ladder (Thermo Scientific GeneRuler 100 bp DNA ladder). In addition, the PCR products underwent digestion with the HphI restriction endonuclease at 37°C for 3 hours, following the manufacturer’s specification. Following this, the products underwent 2% agarose gel electrophoresis and were stained to enhance the visibility of PCR-restriction fragment length polymorphism (PCR-RFLP) profiles. Differences in the size of the DNA fragment that underwent amplification were observed, serving as an indication of the presence of an SNP. Statistical significance was established through both the chi-square test and Fisher's exact test, with significance defined as p-values ≤0.05. All statistical computations were conducted utilizing SPSS version 27 (Armonk, NY: IBM Corp.).

## Results

This study involved 100 individuals without teeth, categorized into case and control groups. The ages of participants ranged from 42 to 70 years. In the case group of 51 individuals, 27 were male while 24 were female. The residual alveolar ridge bone height of the case group ranged from 8.2 to 14.8 mm, with a mean height of 12.1 mm. Conversely, in the control group, ages ranged from 40 to 70 years. In this group, there were 26 males and 23 females, with residual jaw bone ridge height ranging from 16.2 to 29.3 mm and averaging 21.3 mm (Table 1).

**Table 1:** Study group comparison based on demographic information.

Variables		Case group	Control group
Age range (years)		42-70	40-70
Sex	Male, n (%)	27 (52.9%)	26 (53.1%)
	Female, n (%)	24 (47.1%)	23 (46.9%)
Bone height range (mm)		8.2-14.8	16.2-29.3
Mean		12.1	21.3

DNA extraction was conducted on all 100 samples, and subsequently, RFLP analysis was performed. The genotype was determined for all samples in both the study and control groups, except for two samples in the control group, specifically sample numbers 5 and 10, both exhibiting type I ridges.

**Figure 2:**
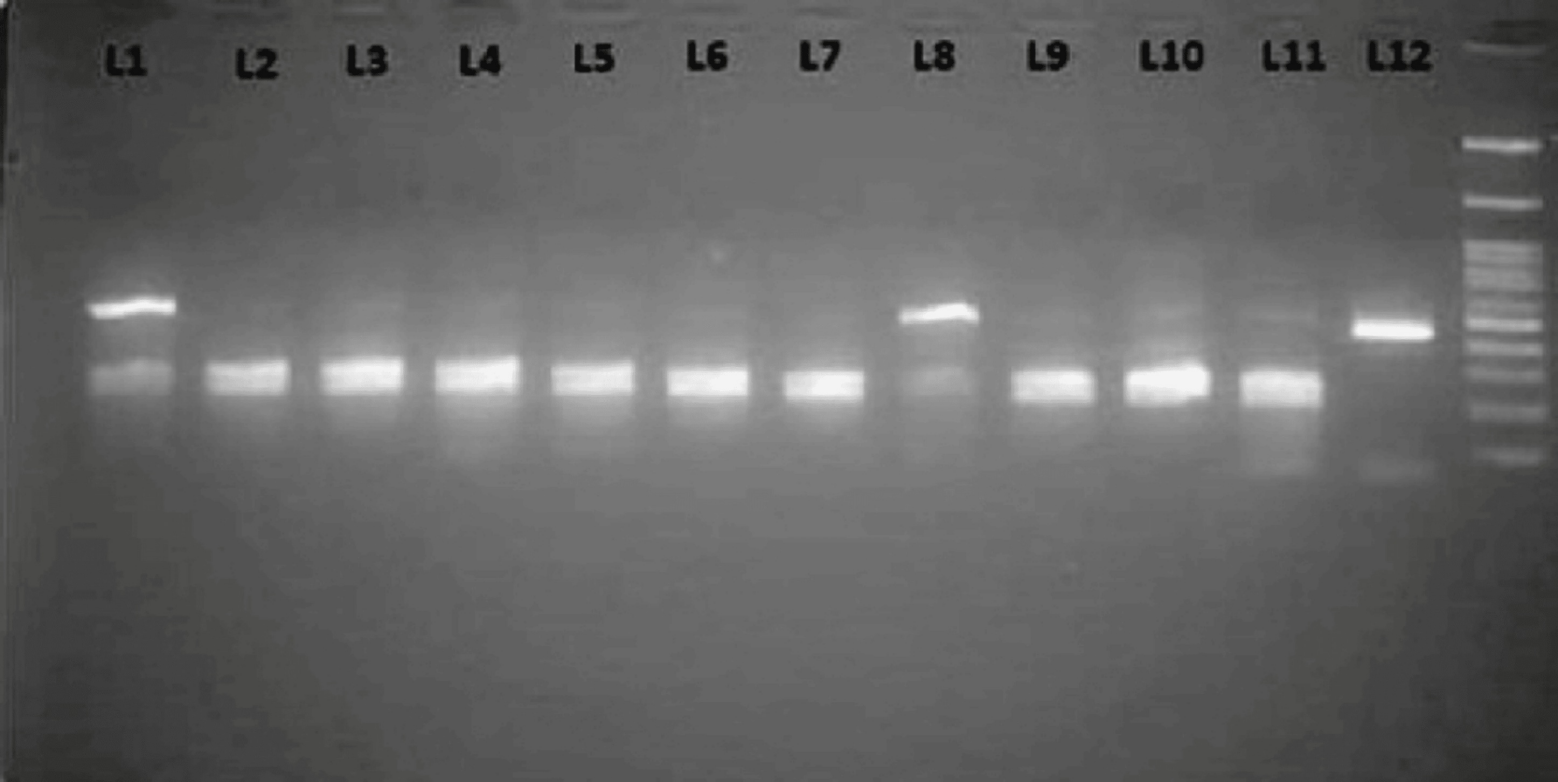
PCR product restriction digestion assay for the HIF1A gene's 1772C>T polymorphism using the HphI enzyme, visualized on a 2% agarose gel. A typical depiction of PCR product restriction digestion for the hypoxia-inducible factor 1 subunit alpha gene's 1772C>T polymorphism, employing the HphI enzyme, was distinguished on a 2% agarose gel. The CT genotype showed three bands (216, 251, 467 bp) in Lanes 1, 8, 10, and 11. The CC genotype showed two bands (216, 251 bp) in Lanes 2-7 and 9. The TT genotype appeared as a single 467 bp band in Lane 12. A 100 bp ladder was included in the last lane for size comparison.

The electrophoretic analysis of a 2% agarose gel revealed the presence of two bands (measuring 251 and 216 base pairs) indicating the C allele. The T allele, on the other hand, produced a single fragment measuring 467 base pairs. The CT genotype, which represents heterozygosity, showed three bands at 216, 251, and 467 base pairs (Lane 1, 8, 10, 11). In contrast, the CC genotype, which indicates homozygosity, showed two bands at 216 and 251 base pairs (Lane 2, 3, 4, 5, 6, 7, and 9). The homozygous genotype TT was observed as a single uncut fragment with a length of 467 base pairs (Lane 12) and is depicted in Figure 2.

**Figure 3:**
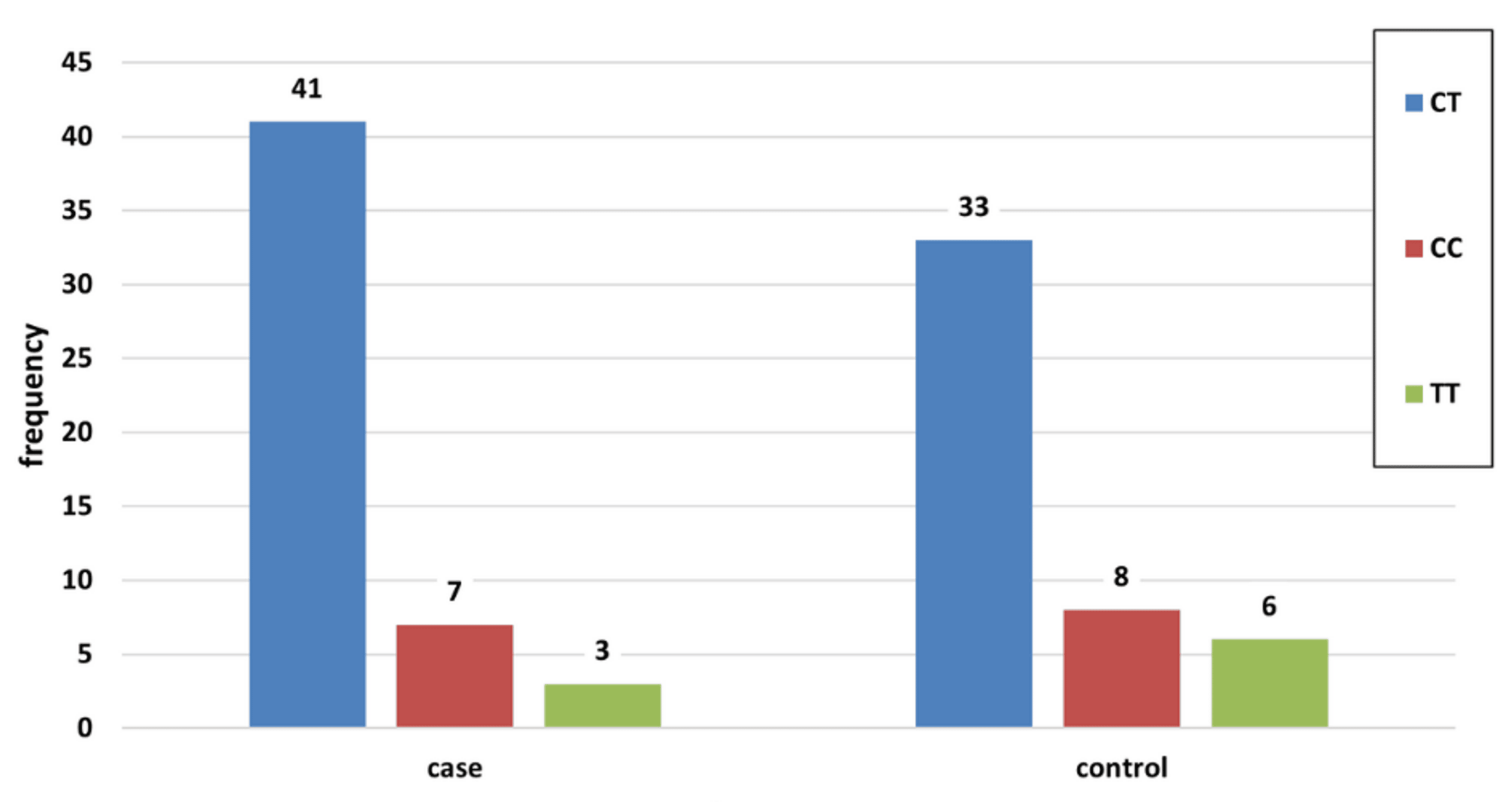
Depicts distributions of genotypes among cases and controls in frequencies.

In the case group, out of the 51 patients experiencing severe residual ridge resorption, 41 cases exhibited the heterozygous genotype CT (80.39%), seven cases showed the homozygous genotype CC (13.73%), and three cases displayed the genotype TT (5.88%), representing homozygosity for the rare allele. Conversely, in the control group, 33 cases presented with the heterozygous CT genotype (70.21%), eight cases with the homozygous CC genotype (17.02%), and six cases with the homozygous TT genotype (12.77%) (Figure 3). Not a single genotype showed statistically significant differences between the groups (CT: chi-square=1.37, p=0.24; CC: chi-square=0.20, p=0.65; TT: Fisher's exact test, p=0.30, SPSS version 27).

## Discussion

This study investigated the relationship between the SNP at position 1772 in the HIF-1α gene and severely resorbed mandibular ridges in fully edentulous patients from the cohort of the South Indian Population. Our sample comprised 100 individuals divided into case and control groups, reflecting the demographic characteristics of the broader population seeking dental treatment in the region. Although no statistically significant disparities in genotype distributions were observed between the case and control groups, our results offer valuable insights into the potential influence of genetic factors on susceptibility to residual ridge resorption. The prevalence of the heterozygous CT genotype in both groups implies a genetic inclination toward residual ridge resorption, though without notable associations were found. Comparison with previous studies, such as research conducted in the Egyptian population, highlights variations in genetic predisposition across different populations [10].

Although our study did not find significant associations, the Egyptian study unveiled a notable variance in the prevalence of the TT genotype, suggesting potential ethnic disparities in genetic susceptibility to jaw bone resorption. Also, the study conducted on Korean subjects examined the genetic association between a specific SNP in the HIF-1α gene and residual ridge resorption with partially or completely edentulous mandibles. Their findings revealed that a minor allele of rs11549467 was significantly associated with residual ridge resorption. This SNP is known to increase HIF-1α transactivity, leading to enhanced angiogenesis and new vessel formation, which may contribute to disturbed bone remodeling balance and subsequent residual ridge resorption [11]. Additionally, research has demonstrated that other SNPs in relevant genes, like fibroblast growth factor receptor 1 oncogene partner 2 (FGFR1OP2)/wt3.0 and vascular endothelial growth factor (VEGF), have been associated with excessive residual ridge resorption in various ethnic groups [12,13]. It is plausible that these genetic variations could also exert an influence on the Indian population [14]. Despite the lack of statistically significant findings, our study underscores the importance of considering genetic factors in residual ridge resorption pathogenesis. The utilization of non-invasive sampling methods and rigorous selection criteria enhances the feasibility and reliability of genetic analysis in routine dental practice, potentially advancing personalized treatment approaches in prosthodontic care not only in South India but also in similar populations globally [15,16].

While our study did not identify significant associations, the observed trends warrant further investigation. Future research endeavors should explore comprehensive genetic profiling encompassing multiple loci implicated in bone metabolism pathways [17]. Longitudinal studies tracking residual ridge resorption progression in conjunction with genetic analyses may elucidate the dynamic interplay between genetic predisposition and environmental factors over time. Limitations of our study include the relatively small sample size and the focus on a specific SNP, which may overlook the contribution of other genetic variants and environmental factors to residual ridge resorption pathogenesis. Additionally, the exclusion of individuals with systemic diseases affecting bone health may have inadvertently excluded subjects with heightened genetic susceptibility to residual ridge resorption.

## Conclusions

While our study did not identify significant associations between a single nucleotide polymorphism found at position 1772 in the HIF-1α gene and significantly resorbed mandibular ridges, it lays the foundation for further investigations into the genetic determinants of residual ridge integrity. By elucidating the intricate interplay between genetic factors and residual ridge resorption pathogenesis, our findings contribute to the evolving landscape of personalized prosthodontic care, fostering the development of targeted interventions to mitigate the adverse sequelae of ridge resorption.
